# Parallel Evolution of *KCNQ4* in Echolocating Bats

**DOI:** 10.1371/journal.pone.0026618

**Published:** 2011-10-24

**Authors:** Zhen Liu, Shude Li, Wei Wang, Dongming Xu, Robert W. Murphy, Peng Shi

**Affiliations:** 1 State Key Laboratory of Genetic Resources and Evolution, Kunming Institute of Zoology, Chinese Academy of Sciences, Kunming, China; 2 Graduate School of the Chinese Academy of Sciences, Beijing, China; 3 Department of Biochemistry, Kunming Medical University, Kunming, China; 4 Centre for Biodiversity and Conservation Biology, Royal Ontario Museum, Toronto, Canada; University of Wyoming, United States of America

## Abstract

High-frequency hearing is required for echolocating bats to locate, range and identify objects, yet little is known about its molecular basis. The discovery of a high-frequency hearing-related gene, *KCNQ4*, provides an opportunity to address this question. Here, we obtain the coding regions of *KCNQ4* from 15 species of bats, including echolocating bats that have higher frequency hearing and non-echolocating bats that have the same ability as most other species of mammals. The strongly supported protein-tree resolves a monophyletic group containing all bats with higher frequency hearing and this arrangement conflicts with the phylogeny of bats in which these species are paraphyletic. We identify five parallel evolved sites in echolocating bats belonging to both suborders. The evolutionary trajectories of the parallel sites suggest the independent gain of higher frequency hearing ability in echolocating bats. This study highlights the usefulness of convergent or parallel evolutionary studies for finding phenotype-related genes and contributing to the resolution of evolutionary problems.

## Introduction

All echolocating bats have the ability to detect and discriminate high-frequency sound. They emit high-frequency calls and use high-frequency hearing to receive echoes from nearby objects, which they can then locate. Indeed, the echolocating bats can detect higher frequencies of sound than non-echolocating bats, which possess the same high-frequency detection ability of most mammals [1]. Although all echolocating bats share this important ability, they do not share a most recent common ancestor. Bats are subdivided into the suborders Yinpterochiroptera and Yangochiroptera based on overwhelming molecular evidence. The former group contains non-laryngeal echolocating Old World fruit bats (family Pteropodidae) plus the superfamily Rhinolophoidea, which uses laryngeal echolocation. The Yangochiroptera includes all other laryngeal echolocating bats [2], [3]. Therefore, the apparent paraphyly of echolocating bats inspires an investigation into the genetic basis of high-frequency hearing. For example, *prestin* is involved in high-frequency hearing and its gene tree groups all echolocating bats together. Moreover, parallel evolution at specific sites occurs in echolocating bats and whales, as well as within echolocating bats [4]–[7]. Given the complex nature of high-frequency hearing, it is likely that other genes are involved in high-frequency hearing in the echolocating bat owing to the complex critical role it plays.

*KCNQ4*, another hearing gene, encodes a protein that forms a voltage-gated potassium channel for the regulation of electrical signaling. In mice, *KCNQ4* is expressed in the basolateral membrane of the outer hair cells and more prominently at the basilar region of the cochlea [8]. The protein pumps potassium outside the cell to bring the cell back to an excitatory condition; new sound stimuli cause the cells to be depolarized once again [9]. *KCNQ4* has 16 exons and the protein contains six transmembrane domains named S1–S6, respectively. These domains are linked by intracellular and extracellular loops flanked by cytoplasmic N- and C-termini. Interestingly, *KCNQ4* is required for high-frequency hearing because mutations of *KCNQ4* in humans can cause non-syndromic DFNA2, the progressive loss of high-frequency hearing [10], [11]. In mice, disruption of the KCNQ4 channel causes them to mimic human non-syndromic DFNA2 [12].

In this paper, we report on the identification of the coding regions of *KCNQ4* from 12 species of echolocating bats and three non-echolocating bats. Analyses of these data discover parallel evolution of *KCNQ4* in the two independent lineages of echolocating bats. We identify parallel evolved sites and trace their historical trajectories in bats.

## Results and Discussion

### Parallel Evolution of *KCNQ4* in Echolocating Bats

**Figure 1:**
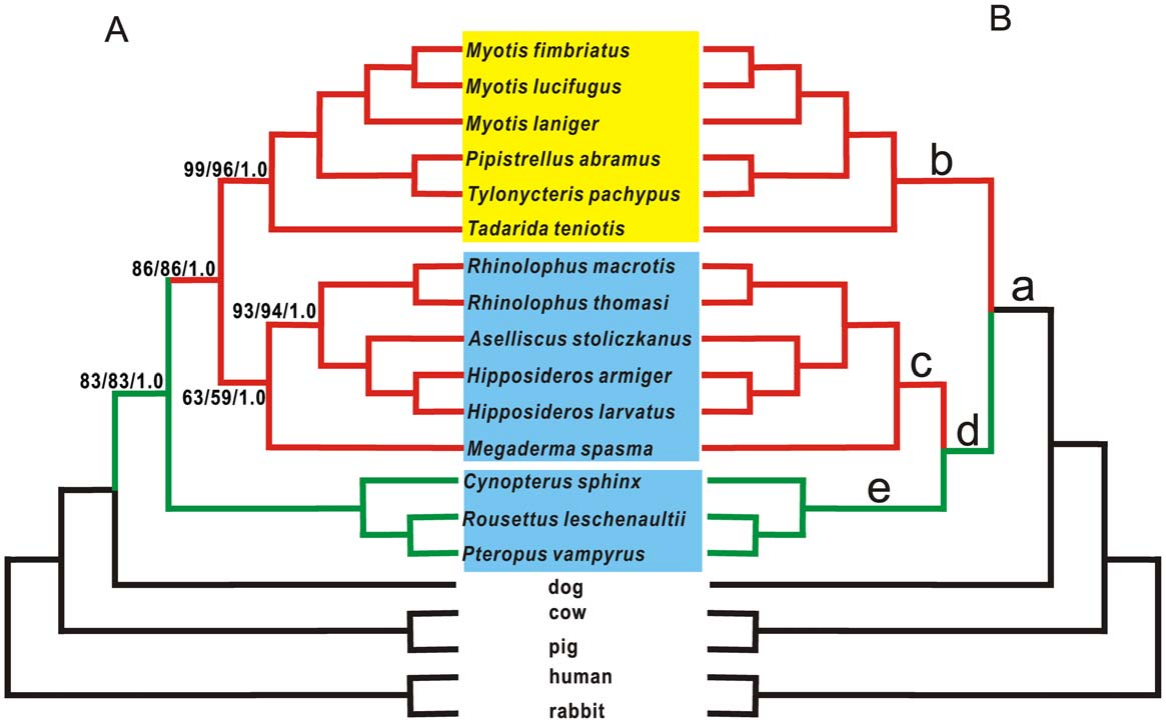
(A) Putative phylogeny based on amino acid sequences of KCNQ4. Values on the branch indicate support from maximum parsimony (MP), maximum likelihood (ML), and Bayesian inference (BI), respectively. The green and red branches indicate echolocating and non-echolocating bats, respectively. The yellow and blue boxes show bats in the suborders of Yangochiroptera and Yinpterochiroptera, respectively. (B) Putative gene tree for KCNQ4 nucleotide sequences using MP and ML. The topology is consistent with that of species tree and bootstrap values are not shown. The letters on the different branches indicate the targets in our selection tests.

We sequenced about 1815 nucleotides of the coding regions of *KCNQ4* from 13 species of bats. The region started with exon 2 and extended to the last exon. We also obtained data for the same region from the draft genome sequences of two additional species of bats (*P. vampyrus* and *M. lucifugus*) and five other mammals. The gene tree for *KCNQ4* was inferred from amino acid sequences using maximum parsimony (MP), maximum likelihood (ML), and Bayesian inference (BI). All methods generated similar topologies (Figure 1A). Bats grouped together with high bootstrap (BS) values of 78–83% and Bayesian posterior probabilities (BPP) of 100%. However, the relationships among non-bat mammals and between non-bat mammals and bats were weakly supported (BS<50%; BPP<0.5). All laryngeal echolocating bats with higher frequency hearing grouped together with strong support (BS = 86–88%; BPP = 100%), rather than being paraphyletic as in the species tree [2], [3]. Comparatively, the Old World fruit bats occupied the most distant position in the topology of the KCNQ4 protein tree (Figure 1A). This topology was unlikely to have been a random resolution because the tree differed significantly (*p*<0.05) from random trees generated by the program Evolver [13]. Further phylogenetic analyses of the nucleotides encoding *KCNQ4* produced topologies consistent with the species tree (Figure 1B).

The conflicting topologies reconstructed from the amino-acid and nucleotide sequences could have resulted from outgroup choice, long-branch attraction, long-branch repulsion and/or heterogeneous base composition [14], [15]. Therefore, we investigated whether any of these factors contributed to the topology of the KCNQ4 tree or not. The topological association of echolocating bats did not change when randomly using species of mammals exclusive of bats as the outgroup. Further, long-branch attraction/repulsion has been suggested to be a problem when unequal or rapid evolution in MP analyses only, or when alignment errors exist in methods based on molecular models [14]. All methods obtained consistent results and the largest pairwise distance for *KCNQ4* sequences was only 0.08. The alignment was unambiguous due to the absence of indels. These observations suggested that the evolution of *KCNQ4* was highly conserved in mammals and, thus, long-branch attraction/repulsion was extremely unlikely. Trees were constructed using sites with synonymous vs. non-synonymous mutations to exclude the heterogeneous base composition as an explanation for the inconsistent trees. Whereas the topology using synonymous mutations was consistent with that of bat species tree (Figure S1), the topology from non-synonymous sites agreed with the KCNQ4 protein tree.

The clustering of echolocating bats in the KCNQ4 protein is not an artifact of data analysis but rather reflects either convergent or parallel evolution. Notably, the topology of the KCNQ4 protein tree is consistent with that of the prestin tree [4]–[7]. This correspondence implies that *KCNQ4* is involved in high-frequency hearing of bats. Further, the comparison of topologies between the functional gene tree and the species tree might be a useful method to identify additional phenotype-related molecular elements, which cluster together when using sequences by function rather than ancestry [16]. Using this method, Li et al. [4]–[5] and Liu et al. [6]–[7] identify the high-frequency gene *prestin* in echolocating bats by reconstructing gene tree which clusters echolocating bats together. Similarly, Castoe et al. [17] analyze the mitochondrial genomes of snakes and agamid lizards and discover that adaptive pressures on metabolic function may drive convergence.

### Identification of Parallel Sites in Echolocating Bats

A ML evaluation [5] was used to identify sites in the KCNQ4 sequences responsible for the anomalous trees. The test discovered a minimum of two sites (476 and 494) that had to be removed to make the likelihood of the species tree higher than that of the KCNQ4 protein tree. All bats with higher frequency hearing in the Yinpterochiroptera and Yangochiroptera shared the same amino acid at these two sites, 476I and 494S. To test the reliability of this method, we deleted both sites and reconstructed the phylogeny from the remaining KCNQ4 protein amino acid sequences. The topology was identical to that of species tree (data not shown).

To determine whether these changes resulted from convergent or parallel evolution in echolocating bats, we inferred the ancestral sequences of KCNQ4 on all interior nodes of the species tree using ML and MP methods [18]. We used the species tree as a basis for constructing ancestral sequences because this tree provided a more reliable evolutionary trajectory than the gene tree. These ancestral reconstructions appeared to be reliable because both methods obtained identical results and the mean BPP exceeded 99% for all nodes of the protein tree. Upon comparing the inferred ancestral sequences of KCNQ4 in all interior nodes of the species tree with the extant sequences, we discovered that these two sites experienced parallel rather than convergent evolution. Statistically [19], parallel changes in laryngeal echolocating bats from the Yinpterochiroptera and Yangochiroptera were significantly more likely than their respective random expectations (*p*<0.01).

Three additional sites are observed to have experienced statistically significant parallel changes in the Yangochiroptera and Rhinolophoidea: 535S, 558E, and 615T (*p*<0.01). All five parallel sites are located on the beginning of the C-terminal of the KCNQ4 protein. Sites 558 and 615 are in the A-domain of KCNQ4, an assembly domain for forming tetramers. Sites 476, 494, and 535 are located in the linker sequence between S6 and the A-domain; they might influence the opening and closing of potassium channels [20]–[22].

Four of five parallel sites in KCNQ4 are located on the ancestral branch of the Yangochiroptera but they are dispersed among laryngeal echolocating bats of the Yinpterochiroptera. This distributional pattern is very similar to that of *prestin*, which has four parallel sites on the ancestral branch of the Yangochiroptera and scattered sites in laryngeal echolocating species of the Yinpterochiroptera [6]. The distribution of parallel sites suggests that the evolutionary trajectories of high-frequency hearing in the Yangochiroptera and Rhinolophoidea might differ. This difference might be caused by distinct call types and auditory characteristics due to close relationship between hearing and calls. Some bats (family Rhinolophidae) in Rhinolophoidea possess an auditory fovea and emit a constant frequency (CF) call and other bats (family Megadermatidae) produce multiharmonic signals; the majority bats in the Yangochiroptera emit brief sound signals [23].

### Association of *KCNQ4* Evolution with Higher frequency Hearing in Bats

**Figure 2:**
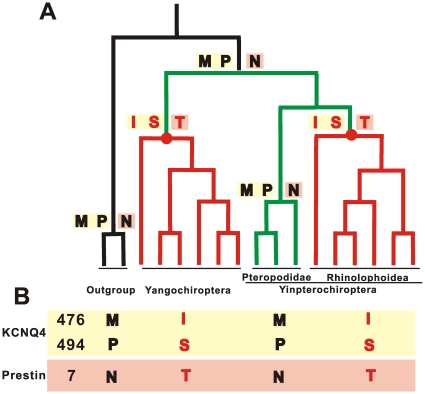
Plot of the number of amino acid changes versus the estimated frequency of best hearing sensitivity. Correlations in the suborder Yangochiroptera and superfamily Rhinolophoidea are significant, respectively (*P* = 0.011, *P* = 0.0065). Triangles indicate the two Old World species of bats which indicate the ancestral states.

Our evolutionary analyses suggested that *KCNQ4* was associated with high-frequency hearing in bats. To further examine this possibility, we used the estimated frequency of best hearing for each species of bat and identified the number of nonsynonymous substitutions leading to that species. We analyzed the relationships between these frequencies and nonsynonymous changes in the Yangochiroptera and Rhinolophoidea, respectively, because two evolutionary processes were involved in high-frequency hearing, as discussed above. A highly significant correlation occurred between the frequencies and number of nonsynonymous changes in the Yangochiroptera and Rhinolophoidea (R = 0.83, *P* = 0.011; R = 0.90, *P* = 0.0065, respectively; Figure 2). The correlations remained significant (R = 0.79, *P* = 0.034; R = 0.83, *P* = 0.042, respectively) after correcting for phylogeny by using an independent contrasts test (PIC) [24], [25].

In contrast to *KCNQ4*, *prestin* does not exhibit a significant correlation between the number of evolutionary changes and best frequencies in bats [6]. A positive correlation does not occur in either the Yangochiroptera (after PIC, *P* = 0.21) or Rhinolophoidea (after PIC, *P* = 0.34). These results reveal that evolutionary changes in KCNQ4 are more closely linked to an increase in hearing sensitivity at higher frequencies in laryngeal echolocating species of the Yangochiroptera and Rhinolophoidea than those in *prestin*. Thus, *KCNQ4* is a better molecular indictor of high-frequency hearing ability in bats than *prestin*.

### Evolutionary Trajectories of Higher Frequency Hearing in Bats

Behavior studies reveal that echolocating bats can detect higher frequency sound than non-echolocating bats, which have the same ability to detect high-frequency sounds as most mammals. This observation raises an interesting conundrum regarding the evolutionary trajectory of high-frequency hearing in bats. *KCNQ4* would be an appropriate indicator to explore this question, as the number of amino acid changes of KCNQ4 exhibits a significant correlation with best frequencies in bats.

We thus estimated the selection pressure of *KCNQ4* gene by comparisons of the rate (ω) of nonsynonymous (amino-acid-replacement) and synonymous (silent) nucleotide substitutions using a likelihood method [18]. Assuming a uniform ω for all branches of the tree for 20 mammalian species (Model 1 in Table 1), ω is estimated to be 0.03, which is not significantly better than the model that allows different ω values for each branch (Model 2 in Table 1). This result suggests that variation of selective pressure among different lineages of mammals is minimal. Similarly, KCNQ4 is under purifying selection on the ancestral branch of all bats (Model 3 in Table 1). We did not find that ω values are significantly different between echolocating bats and non-echolocating bats (Model 4 and Model 5 in Table 1). Further, ω values are significantly smaller than 1 on branch d and e which leaded to the ancestors of Old World fruit bats (Model 6 and Model 7 in Table 1, *P*<0.01 after multiple testing correction), indicating that selection pressure on the ancestral branch of the Old World fruit bats might not be relaxed. Together, these results suggest that KCNQ4 genes are under strong purifying selection in bats and it is very hard to detect the evolutionary trajectory of higher-frequency hearing by testing selective pressures. Purifying selection is not surprising because the genes involved in basic physiological functions, such as hearing, are under functional constrains, as occurs in *prestin* [4].

**Table 1:** Likelihood Ratio Tests of Selective Pressures on mammalian KCNQ4 Genes.

Models	ω (*d_N_/d_S_*)	Ln L	np	Model Compared	2Δ(lnL)	*P* Values
1. All branches have one ω	ω = 0.03	−6673.677	39			
2. Each branch has its own ω	vaiable by branch	−6658.124	75	2 vs. 1	31.106	0.7
3. Branch a has ω_1_ and other braches have ω_2_	ω_1_ = 0.04 ω_2_ = 0.028	−6673.391	40	3 vs. 1	0.572	1
4. Branch b and c have ω_1_, and branch d and e have ω_2_, and other branches each has its own ω	ω_1_ = 0.0572 ω_2_ = 0.0224	−6657.067	74			
5. Branch b and c have ω_1_, and branch d and e have ω_2_ = ω_1_, and other branches each has its own ω	ω_1_ = ω2 = 0.0381	−6657.633	72	5 vs.4	1.132	0.56
6. Branch d and e have ω_1_, and other branches have ω_2_	ω_1_ = 0.0249 ω_2_ = 0.0306	−6673.615	40	6 vs. 1	0.124	0.724
7. Branch d and e have ω_1_ = 1, and other branches have ω_2_	ω_1_ = 1 ω_2_ = 0.029	−6707.513	39	7 vs.6	67.796	9.07E-16*

*Indicates the value corrected by multiple testing (Bonferroni correction).

**Figure 3:**
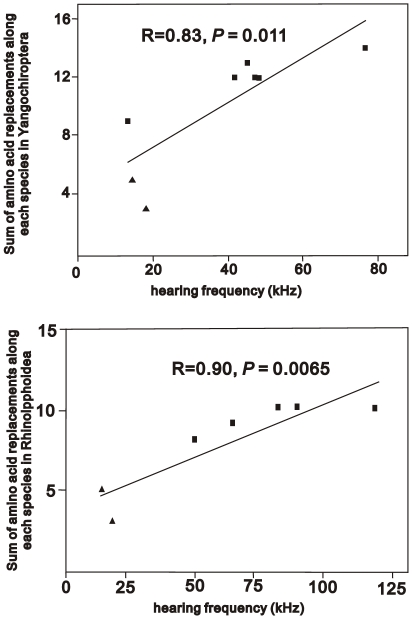
(A) Evolutionary trajectory of parallel sites from prestin and KCNQ4 mapped onto the bat species phylogeny. Red points indicate the nodes where higher frequency hearing might gain. (B) States of parallel-evolved sites from prestin and KCNQ4 on different branches.

However, insights into the generation of higher frequency hearing may be gained by tracing the evolutionary trajectories of the parallel amino acid sites 476 and 494 in KCNQ4. The sites have the same amino acids in all laryngeal echolocating bats with higher frequency hearing. These occurrences are responsible for the grouping of echolocating bats in the KCNQ4 protein tree. A trace of these two amino acid changes on the species phylogeny (Figure 3A) reveals that both sites have the same evolutionary trajectory. Sites 476 and 494 have methionine and proline, respectively, in ancestral bats, a condition identical to that of other extant mammals. However, both sites change into isoleucine and serine, respectively, in the ancestors of the suborder Yangochiroptera and the superfamily Rhinolophoidea. This result suggests that non-echolocating bats retain the ancestral states at sites 476 and 494, as opposed to reversing back to them via additional nonsynonymous mutations (Figure 3B). Thus, the two sites (M476I and P494S) appear to have independently evolved in the ancestors of the Yangochiroptera and Rhinolophoidea. Interestingly, the occurrence of the same parallel evolutionary trajectory in *prestin* also supports this hypothesis (Figure 3A).

Three other sites encoding KCNQ4 appear to have experienced parallel evolution on different branches of the tree for bats with higher frequency hearing: 535, 558, and 615. Moreover, many convergent/parallel/divergent sites are documented for prestin protein sequences on different branches of species tree with higher frequency hearing [6]. This observation suggests that higher frequency hearing in bats evolved subsequent to the origin of bats.

### Conclusions

A high-frequency hearing gene, *KCNQ4*, is identified and, like *prestin*, it appears to have undergone parallel evolution in echolocating bats. The gene tree based on putatively ancestral amino acids is used to identify sites that evolved in parallel. For the first time, the independent gain of higher frequency hearing in bats is found to be based on the parallel evolution of amino acids at functionally associated sites identified for KCNQ4 and prestin. This discovery proposes a hypothesis that higher frequency hearing independently gains at least in the Yangochiroptera and Rhinolophoidea. The occurrence of parallel evolved sites also suggests that higher frequency hearing likely further developed after its origins in bats.

Our results have two important implications. Firstly, the evolution of higher frequency hearing may parallel the evolution of echolocation in bats because it is a precondition for echolocation. Bat echolocation might have independent origins in the ancestors of the Rhinolophoidea and Yangochiroptera. Certainly, additional evidence for this possibility, especially from the fossil record, is required. Secondly, topological comparisons between species and protein trees are a very effective method of discovering trait-related molecular elements, as demonstrated by the high-frequency hearing genes, *prestin* and *KCNQ4*, in bats. Given that we are on the cusp of acquiring thousands of vertebrate genomes [26], this approach may prove to be an exceptionally useful means to identify functional gene complexes.

## Materials and Methods

### Species Coverage, PCR Amplification and Sequencing

We obtained the *KCNQ4* coding region in 15 species of bats having different auditory characteristics. Within the Yinpterochiroptera, representatives of the Old World fruit bats (family Pteropodidae) included *Cynopterus sphinx*, *Pteropus vampyrus*, and *Rousettus leschenaultii*, which do not have laryngeal echolocation. *Megaderma spasma* (family Megadermatidae) can emit short, broadband multiharmonic signals. The leaf-nosed bats, *Hipposideros armiger*, *H. larvatus*, and *Aselliscus stoliczkanus* (family Hipposideridae), and the horseshoe bats, *Rhinolophus thomasi* and *R. macrotis* (family Rhinolophidae), produce constant calls. Species from the Yangochiroptera included *Myotis laniger*, *M. fimbriatus*, *M. lucifugus*, *Pipistrellus abramus*, and *Tylonycteris pachypus* (family Vespertilionidae) and *Tadarida teniotis* (family Molossidae); these species can emit high-frequency echolocation calls with different sound characters [23]. All work with the bats followed Animal Use Protocols approved by the Kunming Institute of Zoology Animal Care and Ethics Committee.

We used RT-PCR to amplify *KCNQ4* from total RNA isolated from brain tissues and cochlear organs in 13 species of bats; we did not sequence *P. vampyrus* and *M. lucifugus* because genomic data were available. For the first-strand cDNA synthesis, 1 µg of total RNA was reverse transcribed in a volume of 20 µl and stored at -80°C for further use. Three pairs of primers (available on request) were designed from conserved sequences of *KCNQ4* from human, dog, cow, horse, *P. vampyrus* and *M. lucifugus* to amplify three overlapping fragments: from exon 2 to exon 7; from exon 6 to exon 14; and from exon 12 to exon 16. Exons 1 of this gene possess high GC content (79.3% in the mouse sequence). We failed to amplify it, even though different primer pairs and different GC-rich buffers were used. All products were isolated from a 1.5% agarose gel and cloned using the T-vector. Positive clones were cycle sequenced in both directions using Big Dye Terminator (ABI) on an ABI3730 sequencer. These sequences were deposited into GenBank (accession numbers: JF826776–JF826788). *KCNQ4* sequences of two species of bats (*P. vampyrus* and *M. lucifugus*), dog (*Canis familiaris*), and rabbit (*Oryctolagus cuniculus*) were obtained from their genomes using our strict protocol [27]. Four splice variants of *KCNQ4* were identified and they did not influence our conclusions regardless of which variant was assessed. We used the longest transcript for the further analysis. *Prestin* sequences were taken from Li et al. [4] and Liu et al. [6].

### Phylogenetic Analysis and Ancestral Construction

To construct a protein tree for KCNQ4, the sequences were first aligned using Clustal W [28]. Tree construction used maximum parsimony (MP) as implemented in MEGA 4 [29], maximum likelihood (ML) using PhyML 3.0 [30], and Bayesian inference (BI) in MrBayes 3.1.2 [31]. For both ML and BI, we used the HIVb+I+G+F amino acid substitution model as evaluated by PROTTEST 2.4 [32]. The ML tree was found by using a heuristic search with five random addition-sequences replicates. ML bootstrap values were obtained from 100 pseudoreplicates from neighbor-joining starting trees. For BI, we set one million generations and a burn-in of 250,000 generations. To compare the topologies of KCNQ4 protein tree and random trees, we generated 1000 random trees for our 20 species by program Evolver in the PAML software package [18].

We used ML methods to locate sites responsible for the topological differences as previously described [5]. In short, we initially calculated the logarithms of likelihood values of the observation of the amino acids at a site given the KCNQ4 protein tree using PhyML 3.0. Similarly, we calculated the logarithms of the likelihood values of the same site given the known species tree. Then, we defined the difference between these two likelihood values from a same site as the d value. We repeated these calculations for every amino acid site and identified the minimum number of sites with the highest d values that needed to be removed to make the likelihood of the species tree higher than that of the KCNQ4 tree. Finally, we reconstructed the protein tree for KCNQ4 after removing these sites and then compared the topologies of the protein and species trees for consistency.

To differentiate between parallel and convergent evolution, we inferred ancestral KCNQ4 protein sequences for each interior node of the 18 species-tree using ML and MP methods of PAML 4.0. Ancestral inferences appeared reliable because mean posterior probabilities for the entire protein exceeded 99% for all nodes. We then looked for either parallel or convergent changes by comparing ancestral and extant KCNQ4 protein sequences. Whereas parallel changes were required to have the same descendant amino acid from the same ancestral amino acid, convergent evolution occurred from a different ancestral amino acid. We calculated the probability that the observed number of parallel or convergent sites exceeded that expected by random chance using the method of Zhang and Kumar [19].

### Tests for Selection

To determine whether or not *KCNQ4* experienced selection in different lineages of bats, we conducted maximum likelihood estimates of the rates of non-synonymous (*d_N_*) and synonymous (*d_S_*) substitutions using the program CODEML [18]. The method identified the kinds of selection involved by comparing the ratio *d_N_/d_S_*, termed ω. Whereas ω<1 indicated purifying selection, ω = 1 indicated neutral selection, and ω>1 adaptive selection.

We conducted a branch-model analysis based on the KCNQ4 gene tree (species tree, Figure 1B) of bats to model selection on different branches. First, we estimated ω under a one-ratio model in which the same ratio occurred across the tree. Second, we estimated an independent ω value for each branch under the free-ratio model, and third, we used the two-ratio ‘branch model’ to compare the estimated ratio on specific foreground branches (ω_1_) in the phylogeny to the background ratio (ω_2_). Branch models were applied to branches that lead to lineages implied to be adapted for higher frequency detection.

### Correlation between Hearing Sensitivity and Amino Acid Changes

To test for a correlation between the frequency of best hearing sensitivity and amino acid changes, we estimated best frequencies using published audiograms. When audiograms were not available, we inferred these values based on call frequencies of maximum energy (Table S1). We then traced and counted all amino acid changes from the common ancestor of bats to each extant taxon. To correct for phylogeny, we conducted an independent contrasts test (PIC) in PDAP [24], [25] based on the species tree with estimated divergent times [3], [33]–[35].

## Supporting Information

Figure S1 **Phylogenetic tree of KCNQ4 inferred from synonymous sites by using Neigbor-Joining method based on Jukes-Cantor model.** Numbers on the branches indicate the bootstrap values>50.
(PDF)

Table S1 Estimated frequency of best hearing sensitivity in bats analyzed in this study.
(DOC)
