# Supplementary material for: Parallel Evolution of KCNQ4 in Echolocating Bats
Source: PLoS One. 2011 Oct 24;6(10):e26618. doi: 10.1371/journal.pone.0026618 (PMC3200345; doi:10.1371/journal.pone.0026618)
Supplement: Figure S1 — Phylogenetic tree of KCNQ4 inferred from synonymous sites by using Neigbor-Joining method based on Jukes-Cantor model. Numbers on the branches indicate the bootstrap values>50. (PDF) [file pone.0026618.s001.pdf]

Figure S1

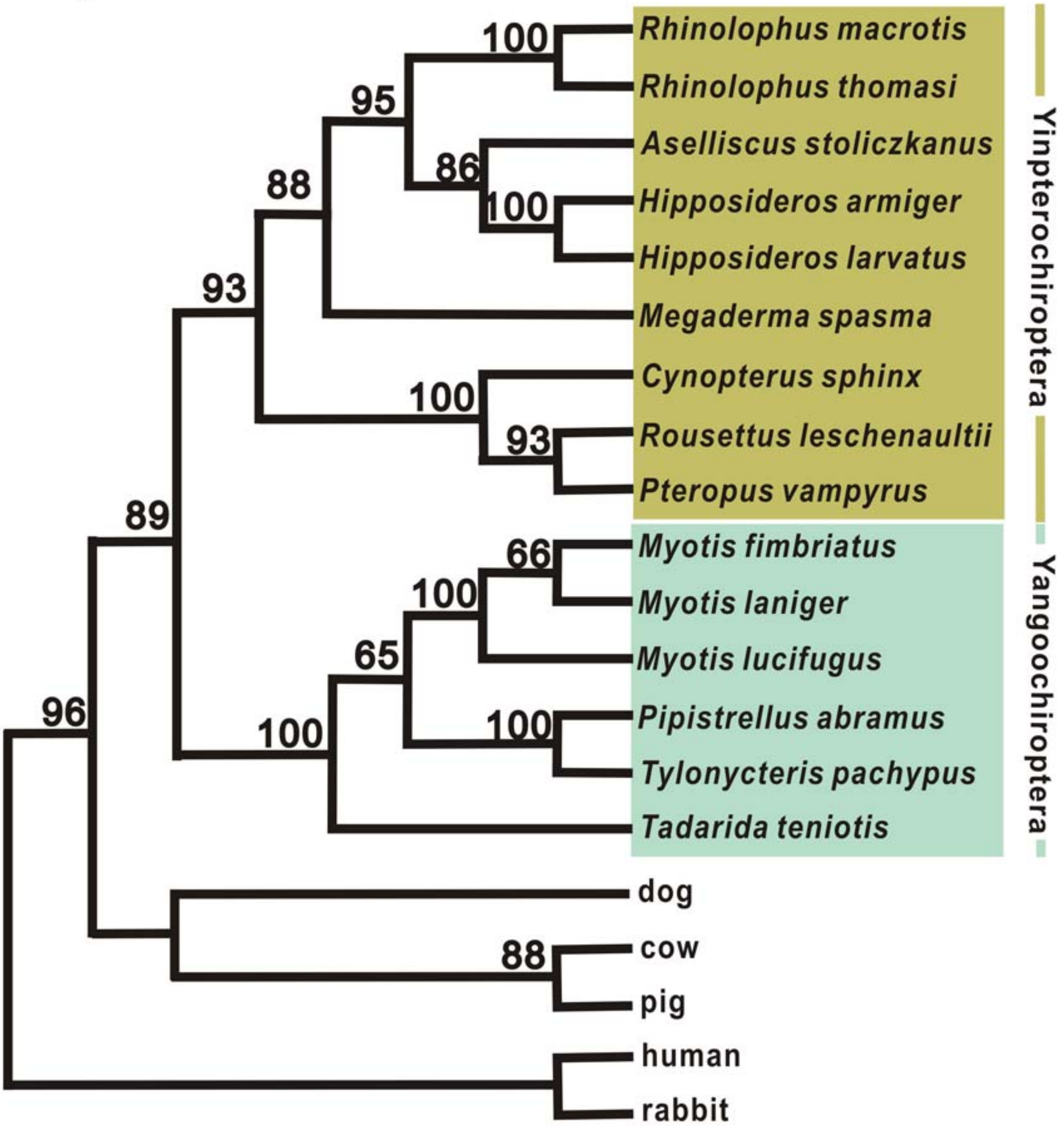

Figure S1. Phylogenetic tree of KCNQ4 inferred from synonymous sites by using Neigbor-Joining method based on Jukes-Cantor model. Numbers on the branches indicate the bootstrap values (not shown below 50)
