# Supplementary material for: Parallel Evolution of KCNQ4 in Echolocating Bats
Source: PLoS One. 2011 Oct 24;6(10):e26618. doi: 10.1371/journal.pone.0026618 (PMC3200345; doi:10.1371/journal.pone.0026618)
Supplement: Table S1 — Estimated frequency of best hearing sensitivity in bats analyzed in this study. (DOC) [file pone.0026618.s002.doc]

Table S1. Estimated frequency of best hearing sensitivity in bats analyzed in this study.

| **Species name** | **Family** | **suborder** | **Estimated frequency of best hearing sensitivity (kHz)** | **Reference** |
| --- | --- | --- | --- | --- |
| *Myotis fimbriatus* |  |  | 51.45 | Liu et al. 2003 |
| *Myotis laniger* |  |  | 45 | http://www.bio.bris.ac.uk/research/bats/ |
| *Myotis lucifugus* | Vespertilionoidea | Yangochiroptera | 47.1 | Dalland 1965 |
| *Pipistrellus abramus* |  |  | 48 | Vater et al. 2003 |
| *Tylonycteris pachypus* |  |  | 76.5 | Zhang et al. 2002 |
| *Tadarida teniotis* | Molossidea |  | 13 | http://www.bio.bris.ac.uk/research/bats/ |
| *Hipposideros armiger* |  |  | 64.8 | Zhang et al. 2009 |
| *Aselliscus stoliczkanus* | Hipposideridae |  | 120.3 | Zhang et al. 2009 |
| *Hipposideros larvatus* |  |  | 82.3 | Zhang et al. 2009 |
| *Rhinolophus macrotis* | Rhinolophoidae | Yinpterochiroptera | 50 | Zhang et al. 2009 |
| *Rhinolophus thomasi* |  |  | 89.7 | http://www.bio.bris.ac.uk/research/bats/ |
| *Cynopterus sphinx* | Pteropodidae |  | 14 | Neuweiler et al. 1984 |
| *Rousettus leschenaultii* |  |  | 25 | Raghuram et al. 2007 |

**References:**

Dalland J. 1965. Hearing sensitivity in bats. Science 150:1185–1186.

Fukui D, Agetsuma N, Hill D. 2004. Acoustic identification of eight species of bat (mammalia: chiroptera) inhabiting forests of southern hokkaido, Japan: potential for conservation monitoring. Zoolog Sci 21:947–955.

Neuweiler G, Singh S, Sripathi K. 1984. Audiograms of a South Indian bat community Journal of Comparative Physiology A: Neuroethology, Sensory, Neural, and Behavioral Physiology 154:133–142.

Vater M, Kossl M, Foeller E, Coro F, Mora E, Russell I. 2003. Development of echolocation calls in the mustached bat, Pteronotus parnellii. J Neurophysiol 90:2274–2290.

Zhang L, Jones G, Zhang J, Zhu G, Parsons S, Rossiter S, Zhang S. 2009. Recent surveys of bats (Mammalia: Chiroptera) from China. I. Rhinolophidae and Hipposideridae Acta Chiropterologica 11:71–88.

Zhang L, Lu L, Zhou S, Dai Q, Zhao H, Lou G, Zhang S. 2002. Comparison of the echolocation signals in two species of flat–headed bats at flying. Zoological Research 23:296–300.
